# Catamenial pneumothorax: a rare condition

**DOI:** 10.23938/ASSN.1076

**Published:** 2024-05-27

**Authors:** Vitorino Modesto dos Santos, Lister Arruda Modesto dos Santos

**Affiliations:** 1 Catholic University Department of Medicine Brasília DF Brazil; 2 Instituto de Assistência Médica ao Servidor Público Estadual (IAMSPE) Advanced General Surgery and Oncosurgery São Paulo SP Brazil

Dear Editor,

In the last number of the journal Anales del Sistema Sanitario de Navarra, Ordóñez Lozano published the case study of 47-year-old woman with moderate chest pain and dyspnea synchronous with menstruation that was diagnosed as a classical catamenial pneumothorax (CP)^1^. She had antecedents of asthma, endometriosis, and the partial resection of the right upper lung lobe due to a spontaneous pneumothorax treated two years ago. The recurrent ipsilateral pneumothorax with persistent air leak was surgically managed by correction of a fissure bleb and numerous millimetric fenestrations in the diaphragm; after an uneventful postoperative course she was discharged to home on the second day^1^. The author emphasized the main physio-pathological hypotheses for a CP development including high prostaglandin F2 levels, transgenital or transdiaphragmatic air passage, micro embolism of endometrial tissue, and tissue migration by retrograde menstruation^1^.

First reported in 1958, CP is a rare recurrent entity (incidence around 1 case per 100,000 inhabitants and year). CP affects females in childbearing age; it associates with endometriosis and can be controlled by hormone therapy or surgery^1^^-^^10^. While CP occurs between 24 h before and 72 h after the onset of the menses, the pneumothorax occurring out of the menstrual period in patients with pelvic or thoracic endometriosis is named endometriosis-related non-catamenial pneumothorax (ER non-CP).

Despite being a well-known entity, during 2023 and 2024 novel studies and clinical cases of CP have been published. Here they are shortly commented aiming to stress the role of current literature on this uncommon entity that frequently poses diagnostic challenges.

In Martinique, 479 cases of pneumothorax from January 2004 to 31 December 2020 were reviewed; 44% of the 212 cases in women were CP, while thoracic endometriosis syndrome (TES) was confirmed in 71 cases including 49 pneumothorax, 14 hemopneumothorax and 8 hemothorax; the incidence of TES was 1.1 cases/100,000 inhabitants/year; mean age at CP diagnosis was 36 ± 6 years, 32 had prior abdominopelvic surgery^2^. Among 71 patients with TES, 69 underwent surgery, 44 (62%) had recurrences in a mean time of 20 ± 33 months; the rates were 84.2% in exclusive medical therapy, 64.7% in only surgery, and 51.8% in patients treated by surgery and medical therapy^2^.

Another single-center retrospective study, performed from January 2011 to December 2020 in France, included 1,284 patients older than 18 years with previously operated pneumothorax. Of them, 331 were women (25.8%), 47 had a CP or ER non-CP (14.2%) and 42 had intra-thoracic endometriosis; 14/47 (30%) had pleural and visceral nodules, 28/47 (60%) had diaphragmatic defects, and 5/47 (10%) had no gross lesions^6^. They were grouped depending on the presence of pleural nodules (N), diaphragmatic perforations (P) or no lesions (NL); seven, twelve and two patients with endometriosis in each group underwent hormonal therapy^6^. The authors emphasized that endometriosis nodular lesions can increase pneumothorax recurrence, and should be resected to prevent prolonged air leaking after the surgery^6^.

A 43-year-old patient with recurrent right-sided chest pain and dyspnea by CP presented pleural effusion, ascites, and left ovarian cyst; thoracoscopy showed hemorrhagic pleural fluid, cysts, nodules and *gunshot lesions* due to pleural endometriosis^2^. She underwent hysterectomy and salpingo-oophorectomy with confirmed endometriosis in the ovary^3^.

A 31-year-old woman with recurrent pleural effusions presented dyspnea and hemoptysis, a pelvic mass and CA-125 high levels; besides episodes of hematemesis during menses^4^. Chest images revealed right pleural effusion, atelectasis, and ascites; a thoracentesis drained two liters of bloody fluid; further video-thoracic procedure evacuated 3.75 liters of hemorrhagic fluid and detected openings in the diaphragm allowing the fluid flow^4^. The pleural implants were biopsied and the immunostaining confirmed the endometriosis; she then underwent a gonadotropin-releasing hormone of depot to prevent the recurrences^4^.

A 34-year-old nulliparous who had dysmenorrhea, presented chest pain and dyspnea during menstrual periods, and there was a right pneumothorax with lung compression, needing a video-assisted thoracoscopic surgery to resect a bulla on the upper lung lobe^4^. With recurrent pneumothorax, and images of uterine fibroids and endometrial polyps, she first used drospirenone and ethinylestradiol, changed to a 19-nortestosterone derivative^5^.

Koike *et al*. reported two cases of CP in Japan. Firstly, a 42-year-old patient with previous endometriosis treated by hormonal therapy, and right sided pneumothorax recurrence^7^. Video-assisted thoracoscopy (VATS) showed endometriosis in diaphragm and pleura; after their resection and placement of an oxidized regenerated cellulose mesh, no recurrence occurred^7^. The other one was a 40-year-old patient with endometriosis treated by hormonal therapy, and recurrent pneumothorax, who underwent VATS to resect multiple endometrial lesions on diaphragm and pleura and similar covering using mesh sheets; no recurren-ces occurred^7^. 

A 21-year-old nulliparous with recurrent right-sided pneumothorax manifested two to three hours prior to the menstrual cycles, was treated by oxygen via a non-rebreather mask, but two weeks later she had recurrent pneumothorax 48 to 72 hours of menses^8^. Pulmonary implants were treated by VATS, and lesions on diaphragm were biopsied; being discharged utilizing norethindrone acetate and gonadotropin-releasing hormone^8^. 

A 38-year-old nulliparous who had an ovarian cyst as a teenager and right pneumothorax at age 35 that was managed by wedge resection, pleurodesis, and diaphragm plication presented intense lower abdominal pain beginning a soon after the menstrual period^9^. Imaging studies revealed a pelvic hematoma in the right adnexal area and large hyperdense right pleural effusion, suggesting passage of blood by diaphragmatic defect^9^. She underwent a laparoscopy which showed approximately one liter of dark red blood and clots in the abdominal cavity, the uterus and adnexa adhered to the colon and pelvic wall, besides lesions resembling endometriosis disseminated throughout the abdomen^9^. The surgical management included lysis of adhesions and irrigation of the hematoma; after hospital discharge under continuous oral contraception for menstrual suppression, the patient was referred to have the regular follow-up in outpatient gynecology service^9^.

A 34-year-old high smoker patient had dyspnea and choking sensation after few days with nausea, vomiting, diarrhea, and abdominal pain, coinciding with her menstruation period; imaging study showed a right-sided pneumothorax, treated ed thoracic tube placement^10^. A month later, the right-sided pneumothorax recurred and she underwent with success a VATS with wedge resection for apical bleb removal, besides the mechanical pleurodesis; however, six months later the chest symptoms recurred associated with menstrual cycle. Reviewing antecedents, she had pelvic endometriosis, but did not use contraceptives; at the recent menses she had right pneumothorax treated by drainage and talc pleurodesis^10^.

The authors strongly believe that publishing single case studies on exceeding uncommon and challenging entities can enhance the awareness and suspicion index of primary care workers and non-specialists, favoring early diagnosis and due management.
